# Impact of two ionic liquids, 1-ethyl-3-methylimidazolium acetate and 1-ethyl-3-methylimidazolium methylphosphonate, on *Saccharomyces cerevisiae*: metabolic, physiologic, and morphological investigations

**DOI:** 10.1186/s13068-015-0206-2

**Published:** 2015-02-08

**Authors:** Nasir Mehmood, Eric Husson, Cédric Jacquard, Sandra Wewetzer, Jochen Büchs, Catherine Sarazin, Isabelle Gosselin

**Affiliations:** Unité Génie Enzymatique et Cellulaire, FRE-CNRS 3580, Université de Picardie Jules Verne, 33 rue Saint-Leu, 80039 Amiens Cedex, France; Unité de Recherche Vignes et Vins de Champagne—UPRES-EA 4707, Université de Reims Champagne-Ardenne, BP1039, 51687 Reims Cedex 2, France; AVT—Biochemical Engineering, RWTH Aachen University, Aachen, Germany

**Keywords:** *Saccharomyces cerevisiae*, Ionic liquid, Second-generation bioethanol, Respirofermentative metabolism, Environmental scanning electronic microscopy

## Abstract

**Background:**

Ionic liquids (ILs) are considered as suitable candidates for lignocellulosic biomass pretreatment prior enzymatic saccharification and, obviously, for second-generation bioethanol production. However, several reports showed toxic or inhibitory effects of residual ILs on microorganisms, plants, and animal cells which could affect a subsequent enzymatic saccharification and fermentation process.

**Results:**

In this context, the impact of two hydrophilic imidazolium-based ILs already used in lignocellulosic biomass pretreatment was investigated: 1-ethyl-3-methylimidazolium acetate [Emim][OAc] and 1-ethyl-3-methylimidazolium methylphosphonate [Emim][MeO(H)PO_2_]. Their effects were assessed on the model yeast for ethanolic fermentation, *Saccharomyces cerevisiae*, grown in a culture medium containing glucose as carbon source and various IL concentrations. Classical fermentation parameters were followed: growth, glucose consumption and ethanol production, and two original factors: the respiratory status with the oxygen transfer rate (OTR) and carbon dioxide transfer rate (CTR) of yeasts which were monitored online by respiratory activity monitoring systems (RAMOS). In addition, yeast morphology was characterized by environmental scanning electron microscope (ESEM).

The addition of ILs to the growth medium inhibited the OTR and switched the metabolism from respiration (conversion of glucose into biomass) to fermentation (conversion of glucose to ethanol). This behavior could be observed at low IL concentrations (≤5% IL) while above there is no significant growth or ethanol production. The presence of IL in the growth medium also induced changes of yeast morphology, which exhibited wrinkled, softened, and holed shapes. Both ILs showed the same effects, but [Emim][MeO(H)PO_2_] was more biocompatible than [Emim][OAc] and could be better tolerated by *S. cerevisiae*.

**Conclusions:**

These two imidazolium-derived ILs were appropriate candidates for useful pretreatment of lignocellulosic biomass in the context of second-generation bioethanol production. This fundamental study provides additional information about the toxic effects of ILs. Indeed, the investigations highlighted the better tolerance by *S. cerevisiae* of [Emim][MeO(H)PO_2_] than [Emim][OAc].

## Background

Depletion of fossil fuels, excess of greenhouse gas, and global planet warming require the exploration of renewable energies that are sustainable from economical, ecological, and environmental points of view [1]. Lignocellulosic plant biomass is generally considered as the most promising renewable feedstock for bioproduction of transportation fuels and commodity chemicals, without alimentary competition [2,3]. Lignocellulosic biomass is composed of three main polymers: cellulose, hemicellulose, and lignin which are tightly linked and organized [1]. For that reason, the bioethanol production from lignocellulosic biomass begins with a pretreatment step to increase cellulose accessibility and digestibility by the cellulolytic enzymes into glucose monomers (improving the saccharification step), followed by an ethanolic fermentation and ends by a distillation step of bioethanol [4].

Several types of pretreatment exist, classified into four categories: chemical, physical, physicochemical, and biological. Chemical pretreatments are the most commonly used for lignocellulosic biomass and include alkali or acid pretreatments, ozonolysis, and organosolv process [1,4].

From the last decade, ionic liquids (ILs) have paid attention in the pretreatment of lignocellulosic biomass under mild conditions with improved yields of reducing sugars [4-7]. ILs are salts typically composed of organic cations and organic or inorganic anions, which exist as liquids at temperatures below 100°C, often at room temperature. They are non-volatile, non-flammable, and present high chemical and thermal stabilities [5-7]. ILs are emerging solvents of interest for lignocellulosic biomass pretreatment, but the complete removal of ILs after pretreatment is technically unrealizable, especially for processes at large scale [8], and residual ILs are still present in the pretreated substrate even after extensive washing [4,5]. Thus, it is essential to investigate their compatibility with all the steps of the bioethanol production process, particularly with the fermentation phase.

Concerning the impact of ILs on microorganisms, conflicting reports are available. Ouellet et al*.* [9] observed that residual 1-ethyl-3-methylimidazolium acetate [Emim][OAc] at a low concentration of 0.1% (v/v) which is remaining after pretreatment of corn stover and switchgrass had significant inhibitory effects on the growth of the yeast *Saccharomyces cerevisiae* and ultimately ethanol production. However Li et al*.* [10] described that corn cob pretreatment with 1-methyl-3-methylimidazolium dimethylphosphite [Mmim][DMP] had no notable effect on enzymatic saccharification, cell growth, and accumulation of lipid of the bacteria *Rhodococcus opacus*. In the same manner, Nakashima et al*.* [11] showed that 1-ethyl-3-methylimidazolium diethylphosphate [Emim][DEP], 1-ethyl-3-methylimidazolium chloride [Emim][Cl], and [Emim][OAc] used for cellulose pretreatments had no negative impact on *S. cerevisiae* growth. Lee et al*.* [12] and Ganske and Bornscheuer [13] observed toxic effects of 1-butyl-3-methylimidazolium hexafluorophosphate [BMIM][PF6] at a concentration of 1% on *Escherichia coli* growth, while no harmful action was found by Pfruender et al*.* [14] at a concentration of 20% on the same bacteria. In another study, Sendovski et al*.* [15] found that [BMIM][PF6] was toxic for *S. cerevisiae*, while no negative repercussion was observed by Pfruender et al*.* [14] with the same microorganism at 20%. Hence, understanding the origin of IL toxicity on cells is gaining interest.

In this work, the impact of two hydrophilic imidazolium-based ILs was investigated on *S. cerevisiae* grown in a culture medium containing glucose as carbon source. The first IL, [Emim][OAc], is commonly used for various lignocellulosic substrate pretreatments, and the second one, 1-ethyl-3-methylimidazolium methylphosphonate [Emim][MeO(H)PO_2_], was recently demonstrated as an efficient alternative to [Emim][OAc] in lignocellulose pretreatment [5,16-19]. The effects of both ILs were observed on the model yeast physiology by following growth, glucose consumption, and ethanol production. Moreover, two novel approaches were used here. The first one is the respiratory activity (oxygen transfer rate OTR and carbon dioxide transfer rate CTR) of yeasts which was followed online by respiratory activity monitoring systems (RAMOS). The second one is the yeast morphology that was observed by the environmental scanning electron microscope (ESEM). To our knowledge, this is the first report of a complete investigation of IL impacts on yeast cells, including physiologic, metabolic, and morphologic parameters.

## Results and discussion

### *S. cerevisiae* growth in the presence of [Emim][OAc] or [Emim][MeO(H)PO_2_] without previous adaptation

**Figure 1:**
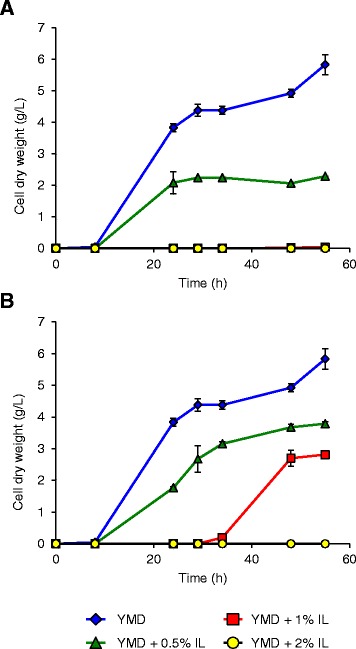
**Growth of non-adapted** ***S. cerevisiae*** **cells in the presence of various concentrations of ILs in YMD culture medium (A [Emim][OAc]; B [Emim][MeO(H)PO**_**2**_ **]).** The results are mean of two experiments, and error bars represent standard deviations from mean value.

The impact of [Emim][OAc] and [Emim][MeO(H)PO_2_] ILs on yeast growth was first assessed by biomass measurements, expressed as cell dry weight (CDW), in YMD culture medium (Figure 1). Without IL, *S. cerevisiae* type II from Sigma-Aldrich grew in YMD medium after an 8-h lag phase to a maximal biomass value of 5.8 g CDW/L (OD_600_ ≈ 13). The [Emim][OAc] IL reduced drastically the growth (Figure 1A): when 0.5% (v/v) was added, the maximum biomass was 2.3 g CDW/L after the same lag phase, and no growth could be observed when 1% or more [Emim][OAc] was added to YMD medium. When [Emim][MeO(H)PO_2_] was added to the YMD medium (Figure 1B), the stationary growth phase was obtained with a biomass value of 3.5 g CDW/L after an 8-h lag phase at 0.5% IL and 2.8 g CDW/L after a 24-h lag phase with 1% IL. Growth was no longer observed with an addition of 2% [Emim][MeO(H)PO_2_] or more (data not shown). The decrease of yeast growth was in agreement with the IL toxic effects already reported in the literature [6,20].

### *S. cerevisiae* adaptation to ILs

In order to increase the yeast tolerance to both ILs, successive cultures of *S. cerevisiae* were realized in YMD supplemented with increasing IL %, separated by a spread on YMD plates (see the “Methods” part). The aim of this protocol was the selection of yeasts with better IL tolerance. This IL-adapted *S. cerevisiae* strain was used for all the further results presented in this study.

### *S. cerevisiae* growth, glucose consumption, and ethanol production in the presence of [Emim][OAc] IL

**Figure 2:**
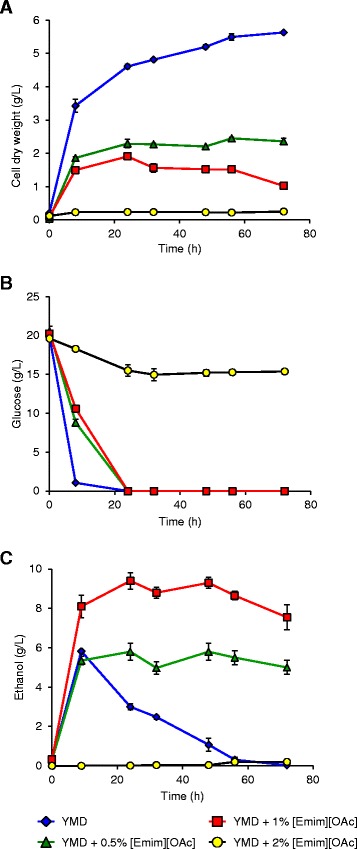
**Growth of IL-adapted** ***S. cerevisiae*** **in the presence of various concentrations of [Emim][OAc] in YMD culture medium (A cell dry weight (g/L); B glucose concentration; C ethanol concentration).** The results are mean of two experiments, and error bars represent standard deviations from mean value.

Figure 2 shows the results obtained with [Emim][OAc] addition to the YMD medium. The maximal biomass was 2.4 g CDW/L with 0.5% IL and 1.5 g CDW/L with 1% IL, both without lag phase. Growth was no longer observed with 2% [Emim][OAc] addition (Figure 2A).

The glucose consumption was measured with an initial concentration of 20 g/L in the YMD medium (Figure 2B) and was proportional to the growth results: glucose was fully consumed for IL addition of 0%, 0.5%, and 1% but poorly decreased with 2% [Emim][OAc].

The ethanol production was also quantified (Figure 2C). Without [Emim][OAc], the measured maximum ethanol yield (5.8 g/L) was obtained at 8 h when glucose was completely metabolized and then ethanol decreased to 0 at 72 h. After glucose depletion, no increase in ethanol concentration was observed which indicated that glucose was the only substrate for ethanol production in YMD medium. The other components of the culture medium, i.e. yeast extract, malt extract, and peptone, could not serve as substrates for ethanol production but however may contribute to the growth of *S. cerevisiae* because the biomass value was 3.4 g CDW/L at 8 h while 5.6 g CDW/L at 72 h.

The addition of [Emim][OAc] modified the ethanol profiles: with 0.5% IL, the observed maximal ethanol concentration was similar to the control without IL (5.8 g/L at 24 h when glucose was totally consumed) but remained constant till the end of the culture (5.0 g/L at 72 h). With 1% [Emim][OAc], the measured maximal ethanol concentration was 9.4 g/L at 24 h when glucose was depleted and showed few variations till 72 h (7.6 g/L).

The constancy of ethanol concentration implied that the ethanol decrease observed for the control without [Emim][OAc] was due to a consumption by the yeasts when glucose was exhausted.

It is worth to notice that the addition of 1% [Emim][OAc] to the fermentation medium allowed to increase the maximal ethanol concentration by a factor 1.6 whereas the yeast growth was reduced by a factor 2.9 (Figure 2A).

### *S. cerevisiae* growth, glucose consumption, and ethanol production in the presence of [Emim][MeO(H)PO_2_] IL

**Figure 3:**
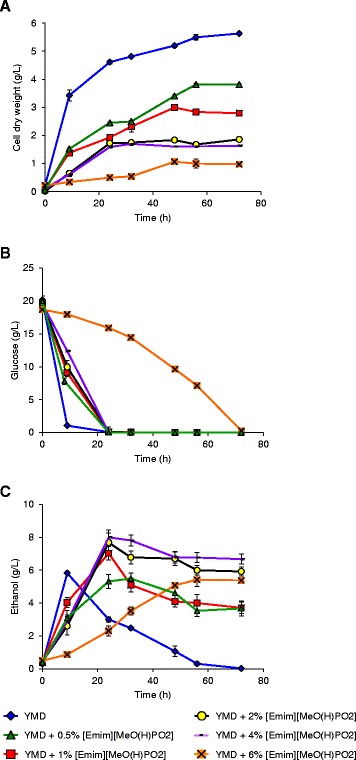
**Growth of IL-adapted** ***S. cerevisiae*** **in the presence of various concentrations of [Emim][MeO(H)PO**_**2**_ **] in YMD culture medium (A cell dry weight (g/L); B glucose concentration; C ethanol concentration).** The results are mean of two experiments, and error bars represent standard deviations from mean value.

Then, the impact of the second IL, [Emim][MeO(H)PO_2_], was investigated on *S. cerevisiae* growth, glucose consumption, and ethanol production. The addition of [Emim][MeO(H)PO_2_] was better tolerated by *S. cerevisiae* than [Emim][OAc] and growth could be observed until a supplementation of 6% IL (v/v) (Figure 3A), indicating that *S. cerevisiae* adaptation to [Emim][MeO(H)PO_2_] was more efficient than to [Emim][OAc]. The maximal biomass decreased progressively with the [Emim][MeO(H)PO_2_] addition from 3.8 g CDW/L (OD_600_ = 8.5) with 0.5% IL (instead of 5.6 g CDW/L for control without IL) till 1.0 g CDW/L with 6% IL. No lag phase was observed except for 6% [Emim][MeO(H)PO_2_]. The totality of glucose contained in the YMD medium was consumed for all the condition tested with a consumption rate inversely proportional to the growth (Figure 3B). The ethanol production was measured (Figure 3C) with a maximum at 5.8 g/L at 9 h for the control without IL followed by a decrease to 0 at 72 h. The [Emim][MeO(H)PO_2_] addition until 6% (v/v) led to a measured maximum ethanol concentration higher than the control with an optimum at 8.0 g/L for 4% [Emim][MeO(H)PO_2_]. Moreover, the decrease in ethanol concentration observed without IL after glucose depletion was less pronounced with [Emim][MeO(H)PO_2_] addition and the ethanol was reduced from 47% at 1% IL, 23% at 2% IL, and 17% at 4% IL between the glucose depletion and the end of the culture at 72 h.

These results showed that both imidazolium-based ILs, [Emim][OAc] and [Emim][MeO(H)PO_2_], increased significantly the measured maximum ethanol yield with 9.4 g/L for an addition of 1% [Emim][OAc] and 8.0 g/L for 4% [Emim][MeO(H)PO_2_] instead of 5.8 g/L ethanol for the control without IL. These values with ILs were closer than the control to the theoretical maximum ethanol yield that could be obtained from glucose at 20 g/L contained in the YMD medium, i.e. 10.2 g/L ethanol [21]. Furthermore, the ethanol consumption by the yeasts after glucose depletion was not observed with ILs addition and instead of a null yield at 72 h for the condition without IL, the ethanol concentrations measured were 7.6 g/L for 1% [Emim][OAc] and 6.7 g/L for 4% [Emim][MeO(H)PO_2_] at 72 h.

The nature of the anion associated to the imidazolium cation seemed to play a deleterious role since the *S. cerevisiae* growth was more drastically affected by the acetate anion (growth until 1%) than by the methylphosphonate one (growth until 6%). But the yeast growth was not correlated to the ethanol production, and the better ethanol yields were obtained with addition of 1% [Emim][OAc] (9.4 g/L ethanol) and 4% [Emim][MeO(H)PO_2_] (8 g/L ethanol) instead of 5.8 g/L ethanol for control without IL.

**Figure 4:**
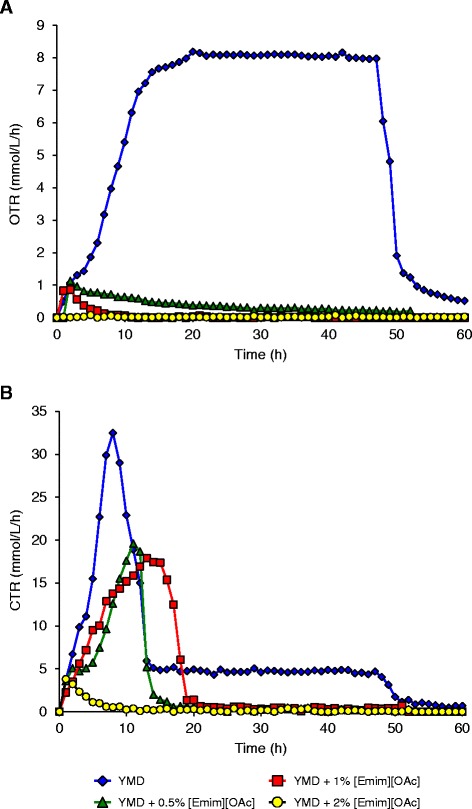
**Oxygen transfer rate OTR (A) and carbon dioxide transfer rate CTR (B) of** ***S. cerevisiae*** **in the presence of various concentrations of [Emim][OAc] in YMD culture medium.** The results are mean of two experiments. Error bars are not represented to avoid overloading the figure.

**Figure 5:**
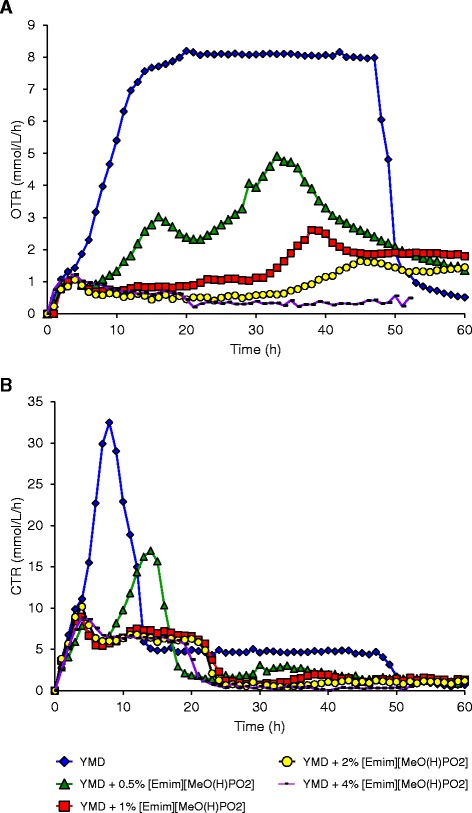
**Oxygen transfer rate OTR (A) and carbon dioxide transfer rate CTR (B) of** ***S. cerevisiae*** **in the presence of various concentrations of [Emim][MeO(H)PO**_**2**_ **] in YMD culture medium.** The results are mean of two experiments. Error bars are not represented to avoid overloading the figure.

As *S. cerevisiae* is a facultative aero-anaerobic yeast, these first results led to investigate the respirofermentative status of the cells which is directly linked to the ethanol biosynthesis [22-25]. For that purpose, the oxygen transfer rate (OTR) was followed during growth in absence or presence of ILs. Secondly, the previous results showed that ethanol was consumed by the yeasts after glucose depletion, underlying an ethanol oxidation into carbon dioxide and water. This hypothesis was verified by measuring the carbon dioxide transfer rate (CTR). Both OTR and CTR were simultaneously followed online during the cultivation time by a RAMOS device proposed by Anderlei and Büchs [26] and Anderlei et al*.* [27]. To our knowledge, this is the first report of OTR and CTR measurements with a RAMOS device applied to yeast grown in presence of ILs. These results are presented in Figure 4 for [Emim][OAc] IL and Figure 5 for [Emim][MeO(H)PO_2_] IL.

### OTR and CTR profiles of *S. cerevisiae* culture in the presence of [Emim][OAc] IL

For *S. cerevisiae* in YMD medium without IL, the OTR increased exponentially at the beginning of the culture from 0 to 7.6 mmol/L/h at 14 h (Figure 4A) and slowly raised to 8.1 mmol/L/h at 20 h. The OTR remained constant until 47 h then suddenly dropped to nearly 0 at 60 h. The OTR increase at the beginning of the culture was correlated with exponential growth phase and was consistent with the carbon substrate consumption [26,28]. The OTR plateau between 20 and 47 h represented the maximum oxygen transfer capacity of the system [27,29] and implied that *S. cerevisiae* fermentation was oxygen limited during this phase. The sharp drop in the OTR at 60 h indicated that the carbon source was completely consumed [26,27].

In the presence of [Emim][OAc], the OTR profiles were different. At 0.5% LI, a small increase was observed at the beginning of the culture from 0 to 1.1 mmol/L/h at 2 h, followed by a slight steady decrease to 0.2 mmol/L/h at 52 h. At 1% LI, the OTR weakly raised to 0.8 mmol/L/h at 2 h, became null at 12 h, and remained constant till the end of the culture. At 2% LI, the OTR value stayed zero during all the experimental time. OTR profiles in the presence of IL concentrations as low as 0.5 or 1% (v/v) showed that [Emim][OAc] greatly modified the oxygen status of the culture and the yeast metabolic behavior. In the absence of [Emim][OAc], glucose was aerobically consumed and converted in ethanol thanks to the short-term Crabtree effect in *S. cerevisiae* which inhibits respiration in the presence of high glucose concentrations and allows aerobic alcoholic fermentation [30-33], thus generating a high OTR maximal value (8.1 mmol/L/h). In the presence of 0.5% and 1% [Emim][OAc], glucose was consumed anaerobically, leading to low OTR maximal values around 1 mmol/L/h [34].

The CTR evolution (Figure 4B) showed for the condition without IL a peak at 32.5 mmol/L/h at 8 h, then a decrease followed by a plateau at 4.8 mmol/L/h from 13 to 47 h, ended by a null value at 50 till 60 h. The first phase of the CTR profile could be correlated with the consumption of glucose converted in carbon dioxide and ethanol until the sugar was depleted at 8 h (Figure 2B, C). Then, the steady CTR at a value well above null between 13 and 47 h implied that another carbon source was consumed by *S. cerevisiae* during that time which was different from the glucose and generating a lower CO_2_ liberation rate. This is consistent with the diminution of ethanol concentration pointed in Figure 2C from 8 to 72 h and showed that *S. cerevisiae* consumed the ethanol produced from glucose during the first phase of the culture [27,34]. When ethanol was totally consumed, the CTR value fell down to 0 at 60 h, underlying that the amino acid nutrients contained in the YMD medium (i.e. yeast extract, malt extract, peptone) could not be used solely by the yeast and that all the carbon sources were exhausted [27].

When 0.5% (v/v) [Emim][OAc] was added to the growth medium (Figure 4B), a CTR peak reaching 19.6 mmol/L/h was observed at 11 h, corresponding to the glucose depletion (Figure 2B); but contradictory to the control condition without IL, the CTR value directly decreased to zero without a plateau around 5 mmol/L/h and stayed null till 52 h. This meant that ethanol was not consumed by the yeast after glucose depletion in that condition and was consistent with the ethanol concentration profile in Figure 2C indicating that ethanol remained constant till the end of the fermentation. At 1% (v/v) [Emim][OAc], the *S. cerevisiae* CTR profile was similar and raised to 17.9 mmol/L/h at 13 h before becoming null at 20 h until the end of the fermentation. With 2% (v/v) [Emim][OAc], the CTR showed a slight peak at 3.8 mmol/L/h at 1 h followed by a decrease to 0 at 10 h and remained constant till the end of the culture at 52 h, which is consistent with the very low yeast growth and glucose consumption observed in Figure 2A, B.

Thus, the maximal CTR value decreased when [Emim][OAc] was added to the medium culture: 32.5 mmol/L/h without IL, 19.6 mmol/L/h with 0.5% IL, and 17.9 mmol/L/h with 1% IL. This indicated that the yeast metabolism had switched from aerobic to anaerobic consumption of glucose with the addition of [Emim][OAc], leading to a lower CO_2_ liberation rate. It is known that anaerobic conditions stimulate ethanol biosynthesis at the expense of biomass production [31,34,35] and could explain the highest ethanol concentration observed in Figure 2C whereas the growth was low (Figure 2A).

### OTR and CTR profiles of *S. cerevisiae* culture in the presence of [Emim][MeO(H)PO_2_] IL

The OTR evolution in presence of the other IL, [Emim][MeO(H)PO_2_], is presented in Figure 5A. The control condition without IL was already described above. When [Emim][MeO(H)PO_2_] was added to the culture medium, the OTR decreased systematically with increasing percentages in IL. With 0.5% IL, the OTR increased from 0 to 1 mmol/L/h at 4 h; then, two OTR peaks were observed, the first at 16 h with 3.0 mmol/L/h and the second at 33 h with 4.9 mmol/L/h. After then, the OTR decreased and reached 1.5 mmol/L/h at 60 h. For conditions with 1% and 2% [Emim][MeO(H)PO_2_], the OTR also increased from 0 to almost 1 mmol/L/h at 4 h and remained constant until 30 h then slightly increased to a maximal OTR value of 2.6 mmol/L/h at 38 h for 1% IL and 1.6 mmol/L/h at 45 h for 2% IL. Then, the OTR decreased and stayed constant to 1.8 mmol/L/h for 1% IL and 1.6 mmol/L/h for 2% IL until 60 h. With addition of 4% [Emim][MeO(H)PO_2_], the OTR increased to 1 mmol/L/h at 4 h and slowly decreased to 0.4 mmol/L/h at the end of the culture.

The effect of [Emim][MeO(H)PO_2_] addition to the culture medium produced the same effect as [Emim][OAc] and induced a transition of the glucose consumption from an aerobic catabolism without IL to an anaerobic behavior in presence of low IL concentrations. But the effect of [Emim][MeO(H)PO_2_] seemed to be less drastic than [Emim][OAc] on *S. cerevisiae*, and the profile obtained with 0.5% [Emim][OAc] was identical to those with 4% [Emim][MeO(H)PO_2_]. This allowed an interesting observation of the transition between aerobic to anaerobic glucose consumption which could be illustrated with the 0.5% [Emim][MeO(H)PO_2_] condition. The OTR profile showed clearly a diauxic growth with two peaks at 16 and 33 h. These two points corresponded to the glucose depletion for the first one (Figure 3B) and to the ethanol consumption for the second one, explaining the decrease in ethanol concentration observed after 32 h in Figure 3C. The diauxic growth with 0.5% [Emim][MeO(H)PO_2_] is also observed in the Figure 3A with a small growth deceleration at 32 h, followed by a second exponential phase until 56 h. When [Emim][MeO(H)PO_2_] percentages increased, the glucose catabolism became more and more anaerobic generating lower yeast growths (Figure 3A) and higher ethanol concentrations (Figure 3C).

The CTR was measured during growth in presence of [Emim][MeO(H)PO_2_] (Figure 5B) and confirmed the previous results. When 0.5% [Emim][MeO(H)PO_2_] was added to the growth medium, the CTR indicated the glucose depletion at 14 h with a peak at 17.0 mmol/L/h and the ethanol consumption at 33 h with an increase to 3.0 mmol/L/h. The ethanol consumption in the second part of the fermentation was less and less observed as the [Emim][MeO(H)PO_2_] percentage raised, for being almost absent in the condition with 4% IL (Figure 5B), exhibiting an ethanol concentration nearly maximal at 72 h (Figure 3C). With 4% IL, the maximal CTR value was 8.8 mmol/L/h observed at 4 h, which is almost four times lower than the control without IL (32.5 mmol/L/h) confirming higher fermentation rates than respiration.

As previously discussed, the two imidazolium-based ILs [Emim][OAc] and [Emim][MeO(H)PO_2_] could decrease the growth, slow down the glucose consumption, and promote ethanol production by inhibiting yeast respiration and avoiding further consumption of ethanol. Then, the impact of both ILs was investigated on *S. cerevisiae* shape and morphology by environmental scanning electron microscopy (ESEM). Here again, to our knowledge, this is the first report of the direct visualization of IL effects on the yeast morphology by electronic microscopy.

### Impact of ILs on *S. cerevisiae* morphology

**Figure 6:**
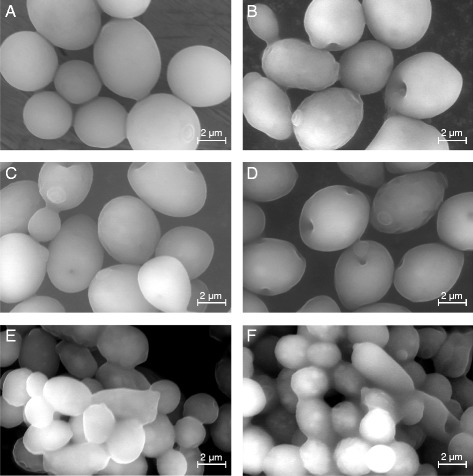
**ESEM micrographs of** ***S. cerevisiae*** **cells in the presence of various concentrations of [Emim][MeO(H)PO**_**2**_ **] in YMD culture medium: (A) YMD; (B) YMD + 1% [Emim][MeO(H)PO**_**2**_ **]; (C) YMD + 2% [Emim][MeO(H)PO**_**2**_ **]; (D) YMD + 3% [Emim][MeO(H)PO**_**2**_ **]; (E) YMD + 4% [Emim][MeO(H)PO**_**2**_ **]; (F) YMD + 5% [Emim][MeO(H)PO**_**2**_ **].**

Figure 6A presents *S. cerevisiae* in the control condition, i.e. YMD medium without IL. The cells were oval, smooth, and swollen, with the typical appearance of ovoid yeasts. Scars from previous buddings could also been observed at the surface of some cells. When [Emim][MeO(H)PO_2_] was added to the culture medium between 1% and 3% (Figure 6B, D), the cell appearance changed: they presented holes clearly visible in the cell wall, some yeasts were wrinkled and exhibited a softer and irregular surface. With higher [Emim][MeO(H)PO_2_] concentrations (4% and 5% in Figure 6E, F, respectively), the yeasts seemed to dissolve and take a gel-like appearance. The cell walls were not clearly distinguishable anymore and cells agglutinated to each other in a sticky and slimy mass.

**Figure 7:**
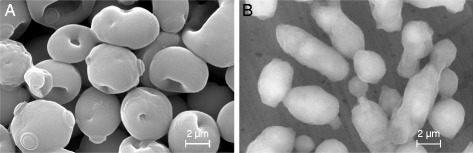
**ESEM micrographs** ***S. cerevisiae*** **cells in the presence of various concentrations of [Emim][OAc] in YMD culture medium: (A) YMD + 0.5% [Emim][OAc]; (B) YMD + 1% [Emim][OAc].**

As already observed, the other imidazolium-derived IL, [Emim][OAc], had the same impact on *S. cerevisiae* morphology (Figure 7), but the deleterious effect was more pronounced and the same damages could be observed with lower IL concentrations. The Figure 7A showed an ESEM micrography of *S. cerevisiae* in the presence of 0.5% [Emim][OAc]. The yeasts presented wrinkled surfaces and exhibited holes in their cell wall, more numerous than with 3% [Emim][MeO(H)PO_2_]. At 1% [Emim][OAc] (Figure 7B), the gelified and smooth aspect of the culture was retrieved. A defect in cell division could also be observed leading to longer cell shapes [36], corresponding to around three or four times the normal yeast length.

These ESEM results showed for the first time the deleterious effects of [Emim][OAc] and [Emim][MeO(H)PO_2_] on *S. cerevisiae* morphology. An explanation could rely on the structure of the yeast cell wall, representing 30% of the cell dry weight and composed of 85% polysaccharides. All the components are linked to each other as a polysaccharide-mannoprotein complex, composed of chains of β-1,3-linked glucose residues branched by β-1,6 linkages, forming a fibrillar glucan serving as a backbone to which is linked chitin, β-1,4-linked N-acetylglucosamine polysaccharide, and some mannoproteins [36,37]. Both imidazolium-based ILs [Emim][OAc] and [Emim][MeO(H)PO_2_] are known and used for their ability to destructure the rigid supramolecular architecture of cellulose, which is a β-1,4-linked glucose polysaccharide [16,17]. It is worth to consider that ILs interacted in a similar manner on the polysaccharides composing the yeast cell wall, inducing holed, softened, and gelified structures as observed in Figure 7. In that context, some authors described ILs as efficient lysis reagents, allowing protein extraction in yeast cells [38]. The IL anionic moiety is describe to be responsible for the disruption of the hydrogen bonds network between the glucosidic monomers in the cellulosic matrix [5,7]. It is probable that the methylphosphonate anion has a lower destructuring ability than the acetate on the yeast cell wall polysaccharides, which could explain the milder deleterious effect of [Emim][MeO(H)PO_2_].

### Impact of ILs on *S. cerevisiae* viability

**Figure 8:**
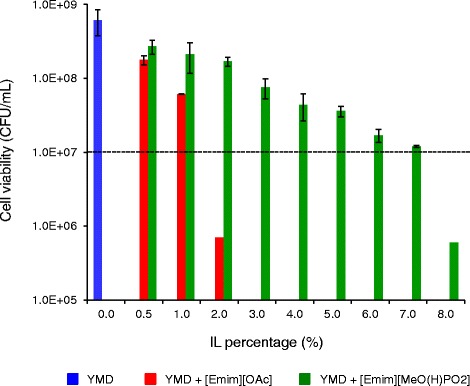
**Viability of** ***S. cerevisiae*** **in the presence of various concentrations of [Emim][OAc] and [Emim][MeO(H)PO**_**2**_ **] in YMD culture medium.** Inoculum was constituted by 10^7^ cells (horizontal dashed line). The colony-forming units were numbered after 24 h of growth.

After visualizing the damages in yeast cell structures due to low concentrations of [Emim][OAc] and [Emim][MeO(H)PO_2_], the cell viability in ILs was quantified by measuring the colony-forming units (CFU) after 24 h of growth in YMD medium supplemented with increasing ILs concentrations. These mixtures were inoculated by 10^7^ cells (Figure 8). In the absence of IL, the *S. cerevisiae* culture contained 6.0 × 10^8^ CFU/mL after 24 h. The addition of 0.5% and 1% [Emim][OAc] was fungistatic and the yeasts grew but to a lesser extent than the control without IL. Cell counts of 1.8 × 10^8^ and 6.1 × 10^7^ CFU/mL, respectively, were reached after 24 h. The [Emim][OAc] became fungicide from 2% (v/v) (7.0 × 10^5^ CFU/mL) and no viable cell could be detected with a 3% addition. The [Emim][MeO(H)PO_2_] was less toxic for *S. cerevisiae* cells: the number of CFU obtained after 24 h of growth was superior to the inoculum until 7% (v/v), indicating fungistatic concentrations. At 8%, [Emim][MeO(H)PO_2_] became fungicide (6.0 × 10^5^ CFU/mL) and no viable cell could be observed from 9% IL (data not shown).

The viability results were consistent with all the previous parts and indicated that [Emim][MeO(H)PO_2_] was more biocompatible with *S. cerevisiae* cells than [Emim][OAc]. It also meant that yeasts retained metabolic activity and reproductive ability, even if they appear damaged with holes in their softened cell walls.

## Conclusions

Altogether, our results showed that both imidazolium-based ILs [Emim][OAc] and [Emim][MeO(H)PO_2_] promoted bioethanol production by *S. cerevisiae*. Addition of low IL concentrations to the growth medium generated higher ethanol yields than the control without IL, i.e. +58.2% with 1% [Emim][OAc] and +37.9% with 4% [Emim][MeO(H)PO_2_]. These increased yields were due to inhibition of the respiration activity of the yeasts, indicated by measured OTR in the culture medium, thereby generating a transition from a respiratory metabolism to a fermentative consumption of glucose with lower biomass production but higher ethanol yields. Moreover, the ethanol consumption by the yeasts observed for the control without IL after glucose depletion was reduced or absent in certain IL conditions. Deleterious effects of ILs were also observed on *S. cerevisiae* cell wall.

The number of ILs is virtually infinite because of unlimited capabilities to exchange constitutive cations and anions. Hence, it is highly probable that new ILs could be designed at will to reduce toxicity and help yeasts to tolerate higher IL concentrations, while retaining the properties of improved ethanolic yields and destructuring the lignocellulosic biomass. But this implies firstly to deepen the knowledge on IL cytotoxicity and how they interact with the cells: some authors pointed the toxic role of the cationic moiety [20,39], others stated for an anionic origin [40], while others evidenced deleterious effects for both anionic and cationic parts [9,41]. Differences between all these reports may originate from the provenance of the ILs, and several works deal with impurities found in commercial ILs that could interact with cells and could lead to distorted toxicity results [42-44].

The future of this fundamental study is to gather the three steps of the second-generation bioethanol production in a one-pot process, regrouping IL pretreatment of the lignocellulosic biomass, enzymatic saccharification, and ethanolic fermentation. A previous study on cellulose pretreatment with [Emim][MeO(H)PO_2_] combined to a simultaneous enzymatic saccharification with *Trichoderma reesei* cellulases showed the best glucose yield with a concentration of 10% [Emim][MeO(H)PO_2_] [17]. We evidenced in our study that *S. cerevisiae* was not viable anymore at that IL concentration and there is more work to do to increase yeast tolerance to ILs and achieve a one-pot process from lignocellulosic biomass to bioethanol, in order to optimize the industrial process and assure a cost-effective ethanolic production [45-47]. This also implies to focus on IL reuse which is still a technological lock even if several works are done in that purpose but only in a separate phase of IL pretreatment [17,18,48]. This will be more difficult to achieve when the ILs are used in diluted media containing enzymes for saccharification and cells for fermentation.

## Methods

### Chemicals

Yeast extract, peptone, and malt extract were obtained from Merck (Darmstadt, Germany) while glucose, osmium tetraoxide (OsO_4_), glutaraldehyde, and agar were purchased from Sigma-Aldrich (Steinheim, Germany). Both ionic liquids, 1-ethyl-3-methylimidazolium acetate [Emim]^+^[CH_3_COO]^−^, and 1-ethyl-3-methylimidazolium methylphosphonate [Emim]^+^[MeO(H)PO_2_]^−^ were acquired from Solvionic SA (Veniole, France), with purity higher than 98%.

### Strain and culture conditions

The yeast strain used in this study was *S. cerevisiae* Type II from Sigma-Aldrich (Steinheim, Germany).

The growth medium was the yeast-malt-dextrose (YMD) medium: glucose 20 g/L, yeast extract 3 g/L, malt extract 3 g/L, peptone 3 g/L, pH 4.8, eventually solidified by agar 20 g/L, and sterilized at 121°C for 20 min. The culture conditions were 30°C at 150 rpm with a shaking diameter of 2.5 cm.

The *S. cerevisiae* strain was conserved at +4°C onto YMD plate. For each experiment, one yeast colony was used to inoculate a 50 mL YMD preculture. At mid-log phase, preculture was used to inoculate the culture at an initial optical density at 600 nm (OD_600nm_) of 0.25 into a 250 mL non-baffled flask containing 50 mL of YMD or YMD + IL from 0.5% to 7% (v/v) added after medium sterilization. The yeast growth was routinely followed by measuring OD_600nm_ values. The biomass was determined as cell dry weight (g/L) after filtration through dried and pre-weighed membrane filter with 0.22-μm pore size (Millipore, Ireland) with subsequent washing in physiological water. The samples were freeze dried and weighted. The CDW was obtained by the difference between the masses of the filter alone and the filter with the yeast cells. All the experiments were realized at least twice.

The yeast viability was measured as number of colony-forming unit (CFU/mL) after sampling a 24-h growth culture, serial dilutions up to 10^−8^ in sterile physiological water, spread of 100 μL of each dilution on YMD agar plates followed by incubation 48 h at 30°C, and counting colonies on plates containing between 30 and 300 colonies.

In order to increase the tolerance of *S. cerevisiae* to both ILs, a series of precultures and cultures was realized in YMD supplemented with increasing IL concentrations (0.5% to 7% v/v). Each run of preculture/culture was separated by a plating on YMD medium, and a colony of this plating was used to inoculate the next run of preculture/culture in increasing IL%. The hypothesis for this adaptation was that only those yeast cells which are resistant to culture with IL% will be able to grow on YMD agar plates. No growth could be observed in culture supplemented with 2% [Emim][OAc] and 8% [Emim][MeO(H)PO_2_], respectively, or higher IL concentrations, even after yeast adaptation. The yeast cells adapted to 1% [Emim][OAc] and 7% [Emim][MeO(H)PO_2_], respectively, were transferred to YMD agar plates. All the further experiments in this study were realized from a colony of these plates.

### Glucose and ethanol quantification

The concentration of glucose was monitored by HPAEC-PAD (high pressure anionic exchange chromatography coupled with pulsed amperometric detection) using an analytical CarboPac PA-20 column kept at 25°C. Elution was carried out at a flow rate of 0.5 mL/min with a gradient method described previously [16,17]. The retention time of glucose was 11.96 min (±0.56%). Quantification was based on calibration curves established using standard glucose.

The ethanol concentration was measured by enzymatic kit from BioLabo SA (Maizy, France).

### Oxygen transfer rate (OTR) and carbon dioxide transfer rate (CTR) measurements

Online measurements of the OTR and CTR in shaken flasks were carried out in a self-made respiratory activity monitoring system (RAMOS) described by Anderlei and Büchs [26] and Anderlei et al*.* [27]. Commercial versions are available from Kühner (Birsfelden, Switzerland) or HiTec Zang (Herzogenrath, Germany). Specially designed flasks were used for cultures in the RAMOS device, but hydrodynamics and gas phase concentrations were similar to those in standard Erlenmeyer flasks with cotton plugs. The measuring cycle was separated into a measuring and a rinsing phase. During the rinsing phase, air with a calculated flow rate flowed through the measuring flask. To protect the measuring flask against contamination, two sterile filters were installed at the inlet and the outlet. The partial pressure of oxygen in the headspace of the measuring flask was detected by an oxygen gas sensor. At the beginning of the measuring phase, the inlet and outlet valves of the measuring flask were closed. The continuing respiration activities of the microorganisms subsequently led to a decrease in the partial pressure of oxygen and to an increase in the carbon dioxide partial pressure in the headspace of the measuring flask. The OTR and CTR of the particular measuring flask were automatically calculated from the change in the partial pressure over time. After the measuring phase, the valves were reopened and “fresh” air could flow through the flask; new measuring cycle started. The measuring cycles were completed approximately in 30 min. A newly developed calibration strategy ensured the compensation of the drift of the oxygen sensors [27]. Due to low solubility of oxygen in culture medium (approximately 8 mg/L at 25°C), it can be assumed that OTR is equal to oxygen uptake rate (OUR). OUR can also be described as the cellular oxygen demand or consumption [49].

### Morphology observations

Microscopic preparations were performed according to a protocol adapted by Gognies et al*.* [50]. The culture samples were immersed in a fixative solution composed of 1% glutaraldehyde in 0.1 M potassium buffer (pH 6.8), at room temperature for 16 h. Samples were subsequently washed with 0.1 M potassium buffer (pH 6.8), post-fixed for 4 h in 2% osmium tetroxide, then washed again with buffer and dehydrated in successive ethanol baths. The morphology of yeasts was observed on a Philips ESEM-FEG XL30 scanning electron microscope in an environmental scanning electron microscope (ESEM) mode.

## Abbreviations

CDW: cell dry weight
CFU: colony forming unit
CTR: carbon dioxide transfer rate
[Emim][OAc]: 1-ethyl-3-methylimidazolium acetate
[Emim][MeO(H)PO_2_]: 1-ethyl-3-methylimidazolium methylphosphonate
ESEM: environmental scanning electron microscope
HPAEC-PAD: high pressure anionic exchange chromatography coupled with pulsed amperometric detection
IL: ionic liquid
OD_600_: optical density at 600 nm
OTR: oxygen transfer rate
RAMOS: respiratory activity monitoring system

## References

[1] Balat M (2011). Production of bioethanol from lignocellulosic materials via the biochemical pathway: a review. Energ Convers Manag.

[2] Naik SN, Goud VV, Rout PK, Dalai AK (2010). Production of first and second generation biofuels: a comprehensive review. Renew Sust Energ Rev.

[3] Sims REH, Mabee W, Saddler JN, Taylor M (2010). An overview of second generation biofuel technologies. Bioresour Technol.

[4] Alvira P, Tomás-Pejó E, Ballesteros M, Negro MJ (2010). Pretreatment technologies for an efficient bioethanol production process based on enzymatic hydrolysis: a review. Bioresour Technol.

[5] Olivier-Bourbigou H, Magna L, Morvan D (2010). Ionic liquids and catalysis: recent progress from knowledge to applications. Appl Catal A.

[6] Quijano G, Couvert A, Amrane A (2010). Ionic liquids: applications and future trends in bioreactor technology. Bioresour Technol.

[7] Mora-Pale M, Meli L, Doherty TV, Linhardt RJ, Dordick JS (2011). Room temperature ionic liquids as emerging solvents for the pretreatment of lignocellulosic biomass. Biotechnol Bioeng.

[8] Zhao H, Jones CL, Baker GA, Xia S, Olubajo O, Person VN (2009). Regenerating cellulose from ionic liquids for an accelerated enzymatic hydrolysis. J Biotechnol.

[9] Ouellet M, Datta S, Dibble DC, Tamrakar PR, Benke PI, Li C (2011). Impact of ionic liquid pretreated plant biomass on *Saccharomyces cerevisiae* growth and biofuel production. Green Chem.

[10] Li Q, Jiang X, He Y, Li L, Xian M, Yang J (2010). Evaluation of the biocompatible ionic liquid 1-methyl-3-methylimidazolium dimethylphosphite pretreatment of corn cob for improved saccharification. Appl Microbiol Biotechnol.

[11] Nakashima K, Yamaguchi K, Taniguchi N, Arai S, Yamada R, Katahira S (2011). Direct bioethanol production from cellulose by the combination of cellulase-displaying yeast and ionic liquid pretreatment. Green Chem.

[12] Lee SM, Chang WJ, Choi AR, Koo YM (2005). Influence of ionic liquids on the growth of *Escherichia coli*. Korean J Chem Eng.

[13] Ganske F, Bornscheuer U (2006). Growth of *Escherichia coli*, *Pichia pastoris* and *Bacillus cereus* in the presence of the ionic liquids [BMIM][BF4] and [BMIM][PF6] and organic solvents. Biotechnol Lett.

[14] Pfruender H, Jones R, Weuster-Botz D (2006). Water immiscible ionic liquids as solvents for whole cell biocatalysis. J Biotechnol.

[15] Sendovski M, Nir N, Fishman A (2010). Bioproduction of 2-phenylethanol in a biphasic ionic liquid aqueous system. J Agr Food Chem.

[16] Husson E, Buchoux S, Avondo C, Cailleu D, Djellab K, Gosselin I (2011). Enzymatic hydrolysis of ionic liquid-pretreated celluloses: contribution of CP-MAS ^13^C NMR and SEM. Bioresour Technol.

[17] Auxenfans T, Buchoux S, Djellab K, Avondo C, Husson E, Sarazin C (2012). Mild pretreatment and enzymatic saccharification of cellulose with recycled ionic liquids towards one-batch process. Carbohydr Polym.

[18] Auxenfans T, Buchoux S, Larcher D, Husson G, Husson E, Sarazin C (2014). Enzymatic saccharification and structural properties of industrial wood sawdust: recycled ionic liquids pretreatments. Energ Convers Manag.

[19] Chen Z, Feng Y, Wang J, Wang J, Guan W, Zhang H (2014). Effects of [C_2_mim][OAc] (1-Ethyl-3-methyl-imidazolium acetate) on the growth of wheat seedlings under Cd^2+^ stress. Bull Environ Contam Toxicol.

[20] Biczak R, Pawłowska B, Bałczewskia P, Rychter P (2014). The role of the anion in the toxicity of imidazolium ionic liquids. J Hazard Mater.

[21] Krishnan MS, Ho NWY, Tsao GT (1999). Fermentation kinetics of ethanol production from glucose and xylose by recombinant *Saccharomyces* 1400(pLNH33). Appl Biochem Biotechnol.

[22] Franzén CJ (2003). Metabolic flux analysis of RQ-controlled microaerobic ethanol production by *Saccharomyces cerevisiae*. Yeast.

[23] Alfenore S, Cameleyre X, Benbadis L, Bideaux C, Uribelarrea JL, Goma G (2004). Aeration strategy: a need for very high ethanol performance in *Saccharomyces cerevisiae* fed-batch process. Appl Microbiol Biotechnol.

[24] Ishtar Snoek IS, de Steensma HY (2007). Factors involved in anaerobic growth of *Saccharomyces cerevisiae*. Yeast.

[25] Rintala E, Toivari M, Pitkänen JP, Wiebe MG, Ruohonen L, Penttilä M (2009). Low oxygen levels as a trigger for enhancement of respiratory metabolism in *Saccharomyces cerevisiae*. BMC Genomics.

[26] Anderlei T, Büchs J (2001). Device for sterile online measurement of the oxygen transfer rate in shaking flasks. Biochem Eng J.

[27] Anderlei T, Zang W, Papaspyrou M, Büchs J (2004). Online respiration activity measurement (OTR, CTR, RQ) in shake flasks. Biochem Eng J.

[28] Stöckmann C, Losen M, Dahlems U, Knocke C, Gellissen G, Büchs J (2003). Effect of oxygen supply on passaging, stabilising and screening of recombinant *Hansenula polymorpha* production strains in test tube cultures. FEMS Yeast Res.

[29] Maier U, Losen M, Büchs J (2004). Advances in understanding and modeling the gas–liquid mass transfer in shake flasks. Biochem Eng J.

[30] Crabtree HG (1929). Observations on the carbohydrate metabolism of tumours. Biochem J.

[31] Baumann K, Dato L, Graf AB, Frascotti G, Dragosits M, Porro D (2011). The impact of oxygen on the transcriptome of recombinant S cerevisiae and P pastoris—a comparative analysis. BMC Genomics.

[32] Christen S, Sauer U (2011). Intracellular characterization of aerobic glucose metabolism in seven yeast species by ^13^C flux analysis and metabolomics. FEMS Yeast Res.

[33] Hagman A, Säll T, Piškur J (2014). Analysis on yeast short-term Crabtree effect and its origin. FEBS J.

[34] Barnett JA, Entian K-D (2005). A history of research on yeasts 9: regulation of sugar metabolism. Yeast.

[35] Frick O, Wittman C (2005). Characterization of the metabolic shift between oxidative and fermentative growth in *Saccharomyces cerevisiae* by comparative ^13^C flux analysis. Microb Cell Fact.

[36] Cabib E, Arroyo J (2013). How carbohydrates sculpt cells: chemical control of morphogenesis in the yeast cell wall. Nat Rev Microbiol.

[37] Lesage G, Bussey H (2006). Cell wall assembly in *Saccharomyces cerevisiae*. Microbiol Mol Biol Rev.

[38] Ge L, Wang XT, Ngin Tana S, Hang Tsaic H, Yonga JWH, Huac L (2010). A novel method of protein extraction from yeast using ionic liquid solution. Talanta.

[39] Thuy Pham TP, Cho C-W, Yun Y-S (2010). Environmental fate and toxicity of ionic liquids: a review. Water Res.

[40] Frade RF, Afonso CA (2010). Impact of ionic liquids in environment and humans: an overview. Hum Exp Toxicol.

[41] Gouveia W, Jorge TF, Martins S, Meireles M, Carolino M, Cruz C (2014). Toxicity of ionic liquids prepared from biomaterials. Chemosphere.

[42] Seddon KR, Stark A, Torres MJ (2000). Influence of chloride, water, and organic solvents on the physical properties of ionic liquids. Pure Appl Chem.

[43] Nockmann P, Binnemans K, Driesen K (2005). Purification of imidazolium ionic liquids for spectroscopic application. Chem Phys Lett.

[44] Urbanek M, Varenne A, Gebauer P, Krivánková L, Gareil P (2006). Determination of trace cationic impurities in butylmethylimidazolium-based ionic liquids: from transient to comprehensive single-capillary counterflow isotachophoresis-zone electrophoresis. Electrophoresis.

[45] Vicari KJ, Tallam SS, Shatova T, Joo KK, Scarlata CJ, Humbird D (2012). Uncertainty in techno-economic estimates of cellulosic ethanol production due to experimental measurement uncertainty. Biotechnol Biofuels.

[46] Konda NVSNM, Shi J, Singh S, Blanch HW, Simmons BA, Klein-Marcuschamer D (2014). Understanding cost drivers and economic potential of two variants of ionic liquid pretreatment for cellulosic biofuel production. Biotechnol Biofuels.

[47] Sarks C, Jin M, Sato TK, Balan V, Dale BE (2014). Studying the rapid bioconversion of lignocellulosic sugars into ethanol using high cell density fermentations with cell recycle. Biotechnol Biofuels.

[48] da Costa Lopes AM, João KG, Rubik DF, Bogel-Łukasik E, Duarte LC, Andreaus J (2013). Pre-treatment of lignocellulosic biomass using ionic liquids: wheat straw fractionation. Bioresour Technol.

[49] Mehmood N, Olmos E, Goergen JL, Blanchard F, Ullisch D, Klöckner W (2011). Oxygen supply controls the onset of pristinamycins production by *Streptomyces pristinaespiralis* in shaking flasks. Biotechnol Bioeng.

[50] Gognies S, Belarbi A, Ait BE (2001). *Saccharomyces cerevisiae*, a potential pathogen towards grapevine, *Vitis vinifera*. FEMS Microbiol Ecol.

